# Using signal-to-cutoff ratios of HIV screening assay to predict HIV infection

**DOI:** 10.1186/s12879-023-08891-9

**Published:** 2023-12-13

**Authors:** Yin-Feng Guo, Shui-Di Yan, Jia-Wen Xie, Mao Wang, Yi-Qiang Lin, Li-Rong Lin

**Affiliations:** 1 Center of Clinical Laboratory, School of Medicine, Zhongshan Hospital of Xiamen University, Xiamen University, Xiamen, China; 2 Department of Basic Medical Science, Xiamen Medical College, Xiamen, China; 3 Institute of Infectious Disease, School of Medicine, Xiamen University, Xiamen, China

**Keywords:** HIV Infection, Signal-to-cutoff ratio, Sensitivity and specificity

## Abstract

**Background:**

The sensitivity of HIV screening assays often leads to a high rate of false-positive results, requiring retests and confirmatory tests. This study aimed to analyze the capability of signal-to-cutoff (S/CO) ratios of HIV screening assay to predict HIV infection.

**Methods:**

A retrospective study on the HIV screening-positive population was performed at Zhongshan Hospital, Xiamen University, the correlation between HIV screening assay S/CO ratios and HIV infection was assessed, and plotted Receiver Operating Characteristic (ROC) curves were generated to establish the optimal cutoff value for predicting HIV infection.

**Results:**

Out of 396,679 patients, 836 were confirmed to be HIV-infected, with an HIV prevalence of 0.21%. The median S/CO ratios in HIV infection were significantly higher than that in non-HIV infection (296.9 vs. 2.41, *P* < 0.001). The rate of confirmed HIV infection was increased with higher S/CO ratios in the screening assay. The ROC curve based on the HIV screening assay S/CO ratio achieved a sensitivity of 93.78% and a specificity of 93.12% with an optimal cutoff value of 14.09. The area under the ROC curve was 0.9612. Further analysis of the ROC curve indicated that the S/CO ratio thresholds yielding positive predictive values of 99%, 99.5%, and 100% for HIV infection were 26.25, 285.7, and 354.5, respectively.

**Conclusion:**

Using HIV screening assay S/CO ratio to predict HIV infection can largely reduce necessitating retests and confirmatory tests. Incorporating the S/CO ratio into HIV testing algorithms can have significant implications for medical and public health practices.

## Background

Acquired immunodeficiency syndrome (AIDS), caused by infection with the human immunodeficiency virus (HIV), is one of the most prevalent sexually transmitted diseases throughout the world [1]. Early and rapid identification of HIV infection is crucial for preventing further transmission and facilitating the timely initiation of appropriate care. Fourth-generation HIV screening tests, which detect both HIV-1/2 antibodies and the P24 antigen, are widely used in clinical, public health, and research settings as screening tests for HIV [2]. However, the high sensitivity of these assays is associated with a high false-positive rate, necessitating retests, and confirmatory tests according to current diagnostic algorithms [3]. This reliance on confirmatory tests may result in missed diagnoses and delayed treatment, posing challenges in terms of cost and human resources.

HIV screening assays are commonly reported as positive or negative based on a signal-to-cutoff (S/CO) threshold. Previous research has shown that the strength of the S/CO value is correlated with the likelihood of a positive confirmatory test result for certain HIV screening assays [4–6]. By adjusting the S/CO threshold, the S/CO ratio of the HIV screening assay may aid in early differentiation between HIV and non-HIV infections, before the results of the HIV confirmatory test are available [5, 7]. However, the prevalence of HIV infection varies across different populations and geographic regions, and different clinical venues use different HIV test platforms, which may limit the generalizability of these findings [8]. Therefore, a reassessment of the S/CO thresholds is necessary to account for methodological variations and population differences across testing institutions before the application of the HIV screening assay S/CO ratio can be effectively used to predict HIV infection.

This retrospective study aims to analyze patients’ data from Zhongshan Hospital, Xiamen University, to evaluate the association between the S/CO ratio and HIV infection within the HIV screening-positive population. Additionally, Receiver operating characteristic (ROC) curves were generated based on the S/CO ratio. The primary objective of this investigation is to establish an optimal cutoff value for predicting HIV infection, thereby enhancing the efficiency of clinical decision-making and treatment processes. This will enable the broader implementation of the S/CO ratio as a predictive tool for HIV infection.

## Methods

### Ethics statement and study population

This retrospective study was conducted at Zhongshan Hospital, Xiamen University, which is an affiliated comprehensive tertiary hospital of Xiamen University. The subjects included inpatients, outpatients and physical examination populations who had undergone HIV screening from January 1, 2016, to December 31, 2022. The inclusion criteria of subjects were available integrity information. No loss of participants occurred during the HIV testing process and no loss of results of tests received by the subjects. A detailed manual chart review was performed to obtain the patients’ clinical characteristics (age, sex, and laboratory data). Ethical approval for this study was obtained from the Research Ethics Committee of Zhongshan Hospital, Xiamen University (xmzsyyky2021-195), per national legislation and the Declaration of Helsinki Guidelines. Adult participants provided written consent to participate and minors provided written assent along with written consent from a parent/legal guardian.

### Diagnosis of HIV infection

In this study, a chemiluminescent immunoassay (Sysmex Corporation, Kobe, Japan) was used as a preliminary screening test for HIV detection. The optical density of the reaction system was measured and compared to the threshold value provided in the manufacturers’ instructions to calculate the S/CO ratio. A S/CO ratio of ≥ 1 was considered a positive result, while a S/CO ratio of < 1 was considered a negative result. If the initial HIV screening assay yielded a positive result, the sample was retested in duplicate using the same chemiluminescent immunoassay. Results were considered HIV-negative if both duplicate tests were negative. If at least one of the tests was positive, the sample was then referred to Xiamen Center for Disease Control and Prevention for further confirmatory using Western Blot (HIV1/2 BLOT 2.2; MP Biomedicals, Singapore). The determination of the positive, indeterminate, or negative status of the tested samples was based on the criteria specified in the manufacturers’ instructions. Indeterminate samples underwent further HIV nucleic acid detection. Determine the test results according to the kit instructions. A “reactive” test result is reported as positive for HIV nucleic acid detection. A “nonreactive” test result is reported as negative for HIV nucleic acid detection. Test results from Western Blot or HIV nucleic acid detection were regarded as the reference standard for diagnosing HIV infection. Finally, HIV screening-positive patients were categorized as HIV-infected if Western Blot or HIV nucleic acid detection was reactive.

### Statistical analysis

The statistical analyses were performed utilizing SPSS Statistics version 26 and GraphPad Prism version 8 (GraphPad Software Inc., USA). Normally distributed data were tested using the Student’s t-test. The Nonparametric Mann-Whitney U test was used when two non-normally distributed groups were compared. Categorical data were tested by Chi-square test and Chi-square test for trend. The Spearman rank correlation test was used to investigate the correlation between two variables. The ROC curve was plotted, the optimal cutoff point was determined using the ROC curve, and the area under the ROC curve (AUC) was calculated. A *P* < 0.05 was considered statistically significant.

## Results

### Characteristics of participants

**Fig. 1:**
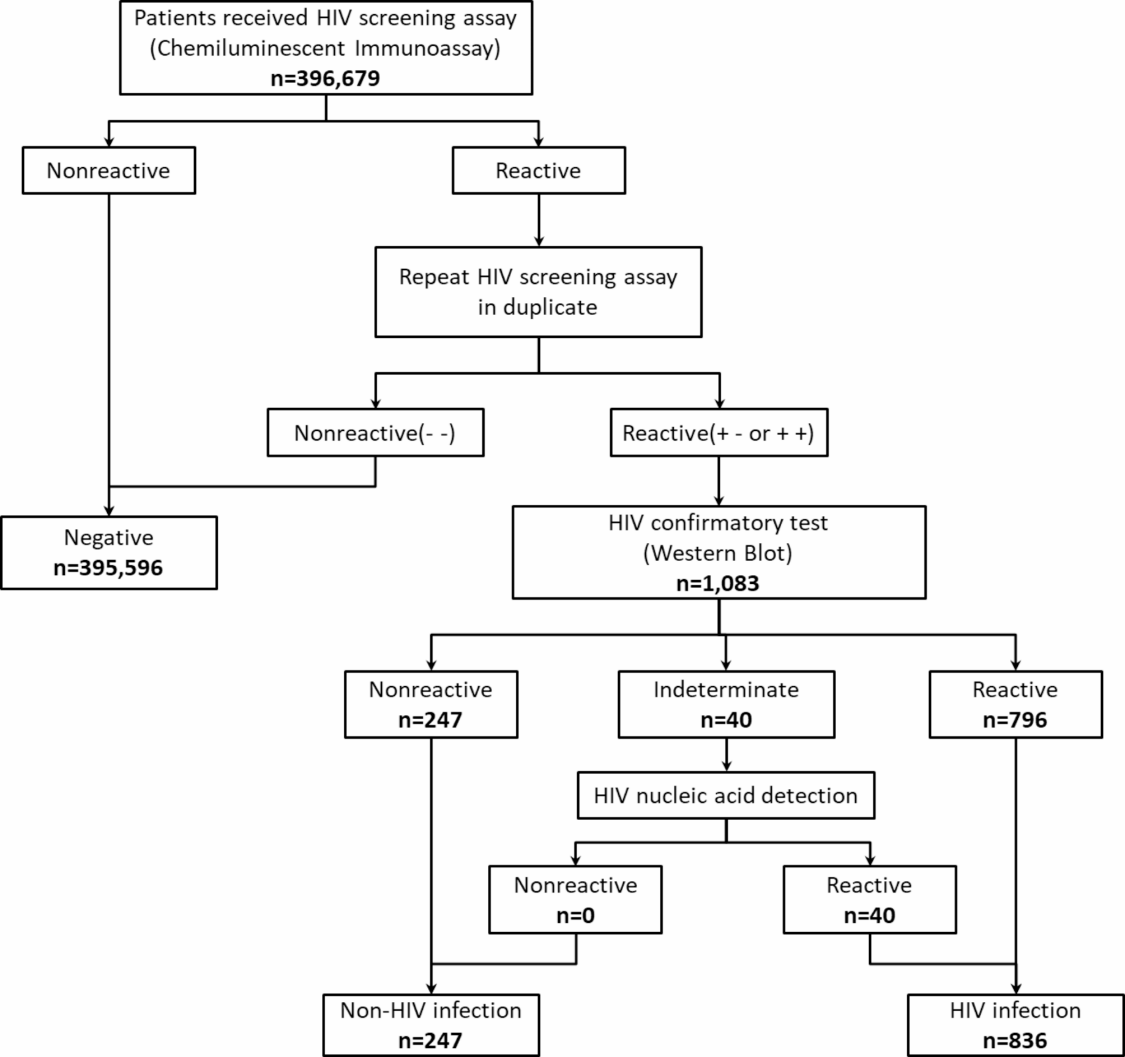
Flowchart showing the steps and results of patients included in the analysis of this study

A total of 396,679 individuals underwent HIV screening assay, with 1,083 patients testing positive for HIV and subsequently undergoing Western Blot confirmatory testing. Of these, 247 patients exhibited a nonreactive result, while 796 patients were reactive. Additionally, 40 indeterminate samples were subsequently serologically reactive. Thus, of the 1083 samples that HIV screening-positive, 836 were confirmed to be HIV-infected, while 247 were not. The HIV prevalence rate was 0.21% (836/396,679) (Fig. 1).

The mean age of HIV infection (39 years) was less than that of non-HIV infection (50 years) (t = 8.532; *P* < 0.001). According to the age group criteria in China and the age distribution of subjects, age was divided into following stages: minor (0–17 years), youth adult (18–28 years), adult (29–40 years), middle age (41–65 years) and old age (≥ 66 years). Comparing the proportion of diagnosed HIV-infected individuals among HIV screening-positive individuals in different age groups, the rate of diagnosed HIV infection among HIV screening-positive patients decreased with increasing age among those aged 18 years and older (χ²=65.347; *P* < 0.001). Comparison of the proportion of HIV-infected patients among HIV screening-positive individuals revealed a statistical difference between the different years (χ² =25.358; *P* < 0.001). This may be related to the different HIV prevalence status and HIV screening populations from year to year. Among HIV screening-positive patients, the rate of HIV infection was higher in males (84.7%) compared to females (51.4%) (χ² =119.384; *P* < 0.001) (Table 1).

**Table 1:** Clinical characteristics of HIV screening positive patients

Characteristic	n	HIV infection(n,%)	Non-HIV infection(n,%)	χ²	*P*
Age(year)				65.347	<0.001
0–17	9	7(77.8)	2(22.2)		
18–28	281	248(88.3)	33(11.7)		
29–40	293	239(81.6)	54(18.4)		
41–65	388	291(75.0)	97(25.0)		
≥ 66	112	51(45.5)	61(54.5)		
Years				25.358	< 0.001
2016	148	128(86.5)	20(13.5)		
2017	151	122(80.8)	29(19.2)		
2018	177	132(74.6)	45(25.4)		
2019	164	110(67.1)	54(32.9)		
2020	150	109(72.7)	41(27.3)		
2021	140	119(85.0)	21(15.0)		
2022	153	116(75.8)	37(24.2)		
Sex				119.384	<0.001
Male	838	710(84.7)	128(12.3)		
Female	245	126(51.4)	119(48.6)		

### Relationship between HIV screening chemiluminescent immunoassay S/CO ratio and HIV infection

**Fig. 2:**
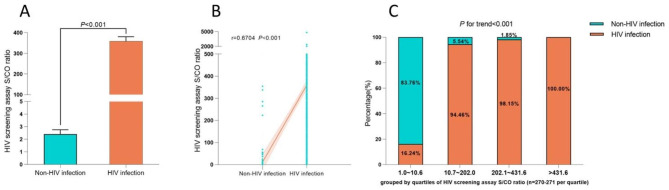
Relationship between HIV screening chemiluminescent immunoassay S/CO ratio and HIV infection. (**A**) HIV screening assay S/CO ratio in the HIV infection and the non-HIV infection. (**B**) Correlation between HIV screening assay S/CO ratio and HIV infection status. (**C**) Correlation of increasing S/CO ratio of HIV screening assay with increasing rate of diagnosed HIV infection

Figure 2 A clearly illustrated the difference in HIV screening assay S/CO ratio between the HIV infection and the non-HIV infection in HIV screening-positive patients. The HIV screening assay S/CO ratio of HIV-infected patients (median: 296.9) was significantly higher than that of the non-HIV infection (median: 2.41) (*P* < 0.001). In Fig. 2B, the Spearman rank test suggested a definite correlation between the HIV screening assay S/CO ratio and the status of HIV infection (correlation coefficient: 0.6704; *P* < 0.001). Then, according to the statistical quartile method, the HIV screening-positive patients were divided into four groups based on HIV screening S/CO ratio: 1.0-10.6 (n = 271), 10.7–202.0 (n = 271), 202.1-431.6 (n = 271), > 431.6 (n = 270), and a Chi-square test for trend was performed to evaluate the association between HIV screening S/CO ratio and the rate of diagnosed HIV infection. The rates of diagnosed HIV infection of groups were presented in Fig. 2C. The Chi-square test for trend revealed a significant correlation between the increasing S/CO ratio of the HIV screening assay and an escalating rate of diagnosed HIV infection (*P* for trend < 0.001).

### The diagnostic capacity of the S/CO ratio of HIV screening chemiluminescent immunoassay to predict HIV infection

**Fig. 3:**
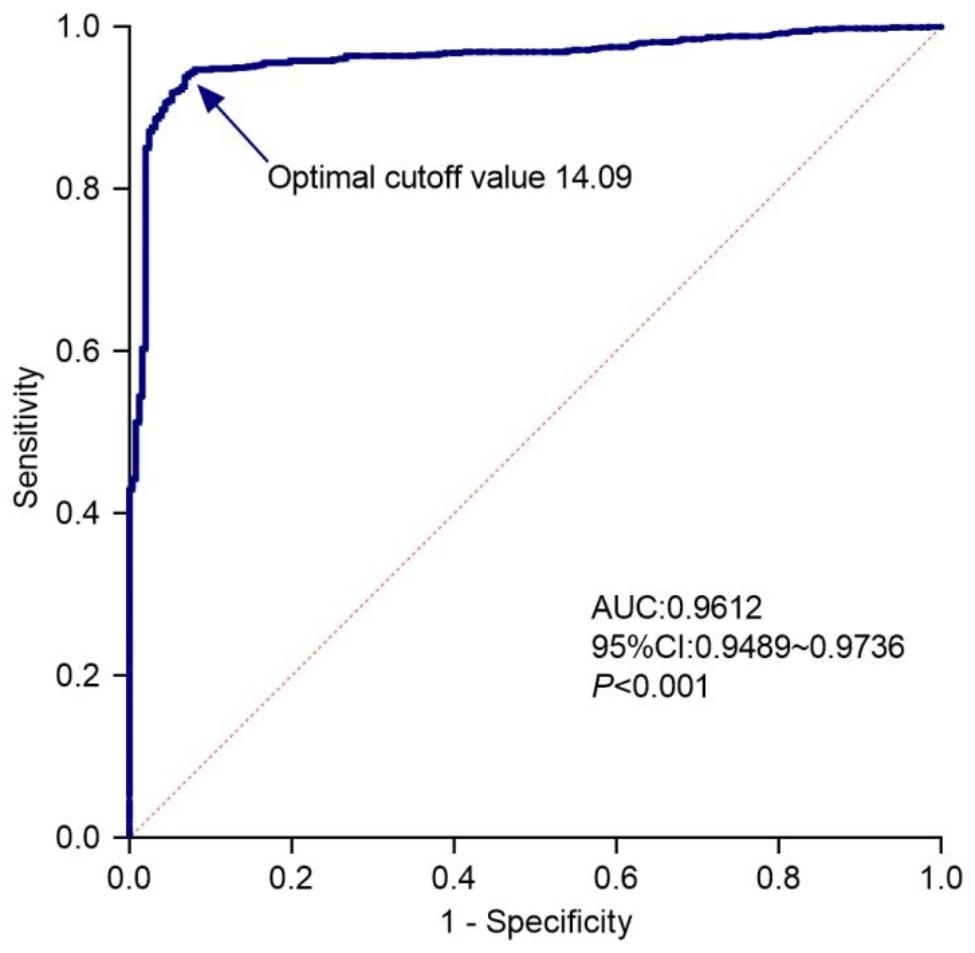
Receiver operating characteristic (ROC) curve analysis for the signal-to-cutoff ratio (S/CO) of HIV screening chemiluminescent immunoassay AUC, Area Under Curve; CI, Confidence Interval

A ROC curve was plotted based on the HIV screening chemiluminescent immunoassay S/CO ratio to evaluate its predictive capability for HIV infection (Fig. 3). The AUC was 0.9612 (95% confidence interval, 0.9489–0.9736; *P* < 0.001). Based on the Youden index, the optimal cutoff value of the S/CO ratio for predicting HIV infection was identified as 14.09, which corresponded with a positive predictive value (PPV) of 97.88%. As the cutoff value of the S/CO ratio increased, the PPV for HIV infection also increased. Further analysis of the ROC curve indicated that the S/CO ratio thresholds yielding PPVs of 99%, 99.5%, and 100% for HIV infection were 26.25, 285.7, and 354.5, respectively (Table 2).

**Table 2:** Cutoff values for predicting HIV infection and corresponding diagnostic indicators in HIV screening positive samples

Cutoff value	Sensitivity(%)	Specificity(%)	Positive predictive value (%)	Negative predictive value (%)
>1.035	100.00	1.62	77.48	100.00
>14.09^*^	93.78	93.12	97.88	81.56
>26.25	90.67	95.55	99.00	75.16
>285.7	51.20	99.19	99.50	37.52
>354.5	42.94	100.00	100.00	34.12

^*^Optimal cutoff value

## Discussion

According to the WHO consolidated guidelines on HIV testing services for a changing epidemic, it is highly recommended that national HIV testing strategies and algorithms abandon the use of confirmatory tests, such as Western Blot and linear immunoassays [9]. These HIV confirmatory tests not only delay the diagnosis and treatment of the disease but also hinder the early detection of HIV infection. To some extent, the strength of the HIV screening assay S/CO value may help the early diagnosis of HIV infection, contributing to replacement confirmatory tests. Thus, an initial objective of this study was to establish a correlation between HIV screening assay S/CO ratio and HIV infection, to assess the ability of the S/CO ratio to predict HIV infection. The results showed that the HIV screening assay S/CO ratio was effective in predicting HIV infection. It was found that HIV screening assay S/CO ratios were significantly higher in HIV-infected patients compared to the non-HIV infected population and that the rate of confirmed HIV infection was increased with increasing HIV screening assay S/CO ratios, which is consistent with previous research [4, 5].

Since 2014, the recommended algorithm for laboratory diagnosis of HIV infection in the United States has consisted of an HIV-1/2 antigen/antibody (Ag/Ab) test followed by an HIV-1/2 antibody (Ab) differentiation test and, if necessary, a diagnostic HIV-1 nucleic acid test (NAT) to resolve discordant or indeterminate results. In China, the “2020 National Guideline for Detection of HIV/AIDS” proposed a new diagnostic algorithm which included nucleic acid detection to resolve indeterminate HIV antibody test results. In this study, we reviewed the HIV test results of 396,679 individuals. HIV nucleic acid detection was performed on 40 patients with indeterminate Western Blot results, all of which were reactive. HIV nucleic acid detection is highly sensitive and can detect the window period of HIV infection in time. Thus, HIV nucleic acid detection can detect acute or early HIV infection, in which HIV antibody tests are likely to be negative or indeterminate [10]. The likelihood of reactive HIV nucleic acid detection results is high in subjects with indeterminate Western Blot results. Man-Qing Liu et al. reported the rate of reactive HIV-1 RNA in indeterminate Western Blot specimens was 67.57%. Specifically, the rate of reactive HIV nucleic acid detection can be as high as 90% in indeterminate Western Blot specimens with double HIV enzyme-linked immunosorbent assays reactive results [11]. In this study, the rate of positive HIV nucleic acid detection in patients with indeterminate Western Blot results was slightly higher than that reported by Man-Qing Liu et al. This may be related to the prevalence status of HIV in different regions. As an inflowing city, the HIV prevalence status of Xiamen is progressively complex because of its numerous migrant populations. When there are more subjects in the acute or early stage of HIV infection, it can lead to the phenomenon of individuals with indeterminate Western Blot results all tested reactive after HIV nucleic acid detection.

Among 396,679 subjects, 1,083 screened positive for HIV, out of which 836 were HIV-infected or living with AIDS. The detection positivity rate of HIV was found to be low (0.21%). In areas with low HIV prevalence, HIV screening is prone to false positive results, which increases reagent and personnel costs [12]. Based on the ROC curve analysis, the optimal cutoff value for predicting HIV infection was the S/CO ratio of 14.09. This threshold corresponds to a PPV of 97.88% and a sensitivity of 93.78%. More than 90% of HIV-infected patients in HIV screening-positive patients can be correctly diagnosed by the S/CO ratio. Although we believe that the highly sensitive screening assays can maximize the identification of potential HIV-infected individuals in low-prevalence areas. Indeed, highly sensitive assays increase the incidence of false-positive results, which translates to a substantial economic burden and the need for human medical resources. The limited human and medical resources emphasize the need for prioritization of beneficial treatments. Therefore, in the face of low HIV prevalence, we hope to utilize HIV screening S/CO ratio to improve the accuracy of predicting HIV infection, which will help to accelerate medical interventions and ensure that truly HIV-infected patients receive treatment while avoiding the negative impact on the large screening populations due to false-positive screening results. According to ROC analysis, we found that the cutoff values with 99%, 99.5%, and 100% PPV for HIV infection were 26.25, 285.7, and 354.5, respectively. Despite a very high PPV, the excessively high cutoff values were extremely low in sensitivity, with a sensitivity of 51.2% for a cutoff value of 285.7 and 42.94% for a cutoff value of 354.5, losing many HIV-infected patients. Thus, we propose to use the S/CO ratio of 26.25 as the cutoff value to predict HIV infection, with 99% PPV and 90.67% sensitivity. Patients with HIV screening assay S/CO ratios greater than 26.25 who undergo confirmatory testing have difficulty in obtaining supplemental information and also delays in accessing treatment and care. Therefore, we recommend informing physicians about the results of HIV screening-positive patients with S/CO ratios greater than 26.25. The physician is also informed that the patient has a very high possibility of HIV infection, but false positives cannot be ruled out. They are advised to make a decision based on the patient’s epidemiologic history and AIDS-related clinical manifestation or to request a confirmatory test.

In our study, more than 80% of men who received positive HIV screening results were eventually diagnosed with HIV infection, while only about half of women were diagnosed with HIV infection, which is consistent with previous studies [6]. In addition, previous studies have shown that the rate of diagnosed HIV infection decreased with increasing age [6, 13], and this finding was also demonstrated in our study. Although the incidence of HIV infection was greater in the 18-28-year-old group than in the 0-17-year-old group in our study, we attribute this to the small sample size in the 0-17-year-old group. Thus, we believe that the accuracy of the HIV screening assay varies for different testing populations with different distributions of S/CO ratios. It may be necessary to implement distinct S/CO thresholds for different testing populations to predict HIV infection. This may be more clinically significant for different testing populations with different behavioral characteristics, such as drug users, men who have sex with men, and sex workers. Unfortunately, due to the lack of information on the behavioral characteristics of the subjects, we are unable to conduct further analysis of HIV screening S/CO ratio thresholds for groups with different behavioral characteristics. Future research may be necessary to unlock and address these queries.

This study also has some limitations. First, the sample source of this study was a single center. The prevalence of HIV infection was inconsistent across regions. The cutoff values used in our study did not apply to other organizations. We recommend that individual institutions research to determine their appropriate cutoff values. Second, for samples with inconsistent results between the HIV screening assay and the confirmatory test, we did not conduct further HIV nucleic acid amplification tests, which may compromise the accuracy in determining the HIV infection status of the samples.

## Conclusions

There is a positive correlation between HIV screening assay S/CO ratios and the likelihood of HIV infection. Using a S/CO ratio of 26.25 to predict HIV infection can largely reduce testing costs. For patients with S/CO ratios higher than this threshold, it can effectively accelerate the time for treatment initiation and enable early intervention. Utilizing the HIV screening assay S/CO ratio to promote updates of HIV testing algorithms will have significant value in medical and public health practice.

## Data Availability

The datasets used and/or analyzed during the current study are available from the corresponding author on reasonable request.

## List of abbreviations

AIDS: Acquired Immunodeficiency Syndrome
AUC: Area under the Receiver Operating Characteristic curve
HIV: Human Immunodeficiency Virus
PPV: Positive Predictive Value
ROC: Receiver Operating Characteristic
S/CO: Signal-to-cutoff
